# Processing Technology Investigation of Loquat (*Eriobotrya japonica*) Leaf by Ultra-Performance Liquid Chromatography-Quadrupole Time-of-Flight Mass Spectrometry Combined with Chemometrics

**DOI:** 10.1371/journal.pone.0064178

**Published:** 2013-05-07

**Authors:** Labin Wu, Xue Jiang, Linfang Huang, Shilin Chen

**Affiliations:** Institute of Medicinal Plant Development, Chinese Academy of Medical Sciences and Peking Union Medical College, Beijing, China; Imperial College London, United Kingdom

## Abstract

Ultra-performance liquid chromatography-quadrupole time-of-flight mass spectrometry (UPLC-QTOF/MS) and multivariate statistical analysis were used to investigate the processing technology of Loquat (*Eriobotrya japonica*) leaf (pipaye, PPY). The differences in samples processed using different methods were revealed by unsupervised principal component analysis (PCA). In the scores plot of PCA, honey-processed PPY (PPPY), crude PPY (CPPY), and heated PPY (HPPY) were clearly discriminated. Furthermore, samples processed at different temperatures could also be distinguished; indeed, our PCA results demonstrated the importance of temperature during processing. Two unique marker ions were found to discriminate between PPPY and CPPY by orthogonal partial least squares discriminant analysis (OPLS-DA), which could be used as potential chemical markers. The method was further confirmed by a verification test with commercial PPY. The orthogonal array experiment revealed an optimized processing condition with 50% honey at 140°C for 20 min after 4 h of moistening time, a process that provides significant information for standardized production.

## Introduction

The leaf of *Eriobotrya japonica* (Thunb.) Lindl (loquat), commonly referred to as pipaye (PPY), is a well-known and commonly used herb in traditional Chinese medicine (TCM). Generally, PPY is used for the treatment of lung-related diseases, including cough, asthma, and chronic bronchitis, as well as for headache, lower back pain, and dysmenorrhea [1]–[3]. Various triterpenes, sesquiterpenes, flavonoids, tannins, and megastigmane glycosides have been identified in PPY, and some of them have been found to possess antitumor, antiviral, hypoglycemic, and anti-inflammatory properties [4]–[8].

In TCM, PPY should be processed before clinical use. According to ancient literature, multiple methods have been used to process PPY. These methods include removing the hair on the leaves, heating [9], and heating in the presence of honey or ginger [10]. Among these methods, removing the hair on the leaves is regarded as a necessary step before using PPY. Today, the honey-heating method is most commonly used as it has been found to be effective in curing cough and pulmonary diseases [11]–[13]. Honey-processed pipaye (PPPY) is used in decoctions and has also been developed as a patent drug in the medicinal market in China. However, chemical analysis and determination of optimal processing mechanisms for PPPY have not yet been investigated, despite the fact that PPPY has been used for hundreds of years. Indeed, although PPPY has been recorded in all versions of the Pharmacopeia of the People’s Republic of China [14], the specific steps and regulatory operations of PPPY have not been established. Therefore, in the current study, we sought to investigate the processing technology of PPY based on chemical analysis and chemometrics. The optimal honey-processing technology of PPPY is also discussed.

Ultra-performance liquid chromatography (UPLC) coupled with photodiode array detector (PDA) and quadrupole time-of-flight mass spectrometry (QTOF/MS) is a newly developed technique that provides a great amount of information rapidly and efficiently compared with other techniques. The high selectivity and sensitivity of UPLC-QTOF/MS makes it a widely applied technique in quantitative and qualitative analysis as well as in metabolite analysis and identification of complex compounds in TCM [15]. To efficiently analyze and compare the information-rich spectroscopic data generated by UPLC-QTOF/MS analysis from different samples, MarkerLynx professional software is often used. MarkerLynx is a peak detection algorithm that analyzes each mass number separately to search for peaks. The area of these peaks would be given an identity of *m/z* and retention times and would then be used as a fingerprint for each sample represented in relation to other samples by PCA. This software provides a repeatable and reliable analytical method for comparing spectroscopic data generated by UPLC-QTOF/MS analysis from 2 or more group samples [16]. In the present study, CPPY, PPPY, and HPPY were analyzed by UPLC-PDA-QTOF/MS coupled with Markerlynx to explore the chemical differences and processing technologies of these different preparations for the first time.

In the chemical analysis of CPPY, HPPY, and PPPY, oleanolic acid (OA) and ursolic acid (UA) are regarded as indicative compounds in evaluation and quality control and are listed as chemical indicators in the Pharmacopeia of China (2010 version) [14]. OA and UA reportedly possess biological activity, including anti-inflammatory [17], [18], antiprotozoal [19], and antimicrobial properties [20], as well as cytotoxicity to cancer cells [21]. OA possesses hepatoprotective [22] and anti-ulcer bioactivities [23], while UA also exhibits antitumor activity through enhancing the production of both nitric oxide and tumor necrosis factor-α via nuclear factor-kappaB activation in resting macrophages [24]. Therefore, in this study, we evaluated the significance of PPY processed using different methods and investigated the optimal processing technology of PPY based on measurement of OA and UA.

## Materials and Methods

### Reagents and plant materials

Reference substances for OA and UA (batch no. OA: 11090502, UA: 12020602, Must Co., Ltd., Sichuan, China); acetonitrile and methanol (CR, chromatographic reagent; Fisher Scientific Co., Ltd., MA, USA); ethanol (AR, analytical reagent) and ammonium acetate (AR; Xilong Co., Ltd., Shanxi, China); and honey (edible sophora flower honey, Baihua Honey Co., Ltd., Beijing, China) were purchased from the indicated companies. Leaves from *E. japonica* (Thunb.) Lindl. were collected from Changshou Feilong (Chongqing, China) on November 20, 2011. Random test samples of *E. japonica* leaves, including crude and honey-processed samples, were from Tong Ren Tang Co., Ltd. (Beijing, China).

### Preparation of samples

First, honey (honey:PPY ratio, 1∶1 w/w) was dissolved in water (50%, v/v). The honey solution was brushed onto the surface of PPY, which was then sealed in a container for 2–4 h. Next, the sample was heated in an oven at 80, 100, 120, 140, or 160°C for 20 min. Samples were then cut into slices (2 mm×30 mm) to yield PPPY slices for analysis. This method has been submitted for an invention patent in China, with a patent application number of 201210384659.2. HPPY samples were heated in an oven at 80, 100, 120, 140, or 160°C for 20 min, respectively.

One gram of each sample was weighed accurately into a conical flask with a stopper. Then, 50 mL ethanol was added to the sample. The solution was extracted ultrasonically (250 W, 50 kHz) for 30 min. The sample solutions were subsequently filtered through a 0.22-μm membrane and then injected into the HPLC and UPLC-QTOF/MS system for analysis [14].

### High-performance liquid chromatography (HPLC) and UPLC-MS methods

The HPLC system model 1525 (Waters, Milford, MA, USA), including binary gradient pump, vacuum degas machine, automatic sample injector, constant temperature column oven, dual wavelength ultraviolet detector model 2487, Breeze chromatographic working station model; chromatographic column model (C_18_ column, 250 mm ×4.6 mm, 5 μm, Waters). For UPLC analysis, the following systems/parameters were used: Waters Acquity system (Waters) equipped with binary solvent delivery pump, auto-sampler, and PDA detector and connected to a Waters Empower 2 data station; Waters Acquity UPLC BEH C_18_ column (2.1 mm ×100 mm, 1.7 μm, Waters); ultrasonication (250 W, 50 kHz, Kunshan Ultrasonic Instrument Co., Zhejiang, China); and an electronic analytical balance model AB135-2 (Mettler-Toledo., Greifensee, Zurich, Switzerland).

OA and UA were analyzed by HPLC. An acetonitrile-methanol-0.5% ammonium acetate solution (67∶12∶21) was set as the mobile phase. The wavelength was set to 210 nm. The references substances, OA and UA, were prepared with ethanol [14]. UPLC separations were carried out in a binary mobile phase at a flow rate of 0.25 mL/min. The optimized separation conditions were as follows: solvent (A), acetonitrile-methanol (5∶1); and solvent (B), 0.5% ammonium acetate. The gradient elutions were as follows: 0–10 min, 70%–80% A; 10–12 min, return to initial conditions. The sample volume injected was 5 μL.

The UPLC/MS analysis was performed on a QTOF Synapt G2 HDMS system (Waters, Manchester, UK) equipped with an electrospray ionization (ESI) source operated in the negative ion mode. N_2_ was used as the desolvation gas. The desolvation temperature was set at 450°C at a flow rate of 800 L/h, and the source temperature was set at 120°C. The capillary and cone voltages were set to 2500 and 40 V, respectively. The data were collected between 50–1200 Da with a 0.1-s scan time and a 0.01-s interscan delay over a 12 min analysis time. Argon (Ar) was used as the collision gas at a pressure of 7.066×10^−3^ Pa. All the MS data were collected using the LockSpray system to ensure the mass accuracy and reproducibility. The [M–H]^−^ ion of leucine-enkephalin at *m/z* 554.2615 was used as the lock mass in negative ESI mode.

### Methodological evaluation

The calibration curve, inter- and intraday precision, repeatability and recovery rates were measured as above (Table 1). The calibration curve and precision were tested with OA and UA; the repeatability and recovery rates were tested with PPY. The calibration curves of OA and UA were Y = 416970×–4012 (r = 0.9997) and Y = 798659x –662112 (r = 0.9991); and the linear ranges of OA and UA were 0.061–1.22 μg and 0.26–5.2 μg, respectively. Limits of detection (LODs) were established at a signal-to-noise ratio (*S*/*N*) of 3. Limits of quantification (LOQs) were established at an *S*/*N* of 10. The LODs of OA and UA were calculated to be 0.9 and 1.0 μg/mL, and the LOQs of OA and UA were calculated to be 2.8 and 3.0 μg/mL, respectively.

**Table 1:** Methodological validation.

				Recovery (N = 6)			
Standard compound	Interday precision (RSD%) N = 6	Intraday precision (RSD%) N = 6	Repeatability (RSD%) N = 6	Mean (%)	RSD (%)	LOD (μg/mL)	LOQ (μg/mL)
Oleanolic acid	0.0363	0.9436	1.1630	97.20	2.12	0.9	2.8
Ursolic acid	0.2332	1.9917	0.8563	98.53	1.10	1.0	3.0

### Data analysis

UPLC-QTOF/MS data for CPPY, HPPY, and PPPY samples were analyzed to identify potential discriminant variables. Peak finding, alignment, and filtering of ES- raw data were carried out with MarkerLynx applications manager version 4.1 (Waters, Manchester, UK). The parameters used were as follows: retention time (t_R_) ranging from 0 to 12 min, mass ranging from 50 to 1200 Da, retention time tolerance of 0.02 min, and a mass tolerance of 0.02 Da. The noise elimination level was set at 6.00, and the minimum intensity was set to 15% of base peak intensity. For data analysis, a list of the intensities of the detected peaks was generated using retention time and mass data (*m/z*). An arbitrary ID was assigned to each of these t_R_–*m/z* pairs with the order of the UPLC elution. The ion intensities for each detected peak were normalized against the sum of the peak intensities within that sample using MarkerLynx software. Ion identification was based on the t_R_ and *m/z*. The resulting 3-dimensional data comprising the peak number (t_R_–*m/z* pair), sample name, and ion intensity were analyzed by PCA and orthogonal partial least squares discriminant analysis (OPLS-DA) in MarkerLynx software [25].

### Orthogonal array design

The orthogonal array design was performed on Orthogonality Experiment Assistant II software (Sharetop Software Studio, 2002, Beijing, China). The influential factors were set as the amount of honey, the heating temperature, the heating period, and the sealing period during the pre-experiment because these factors could affect the attribution of TCMs significantly in others herbs [26]. The levels of these factors are presented in Table 2 according to our pre-experiments and previous literature. The orthogonal array design was performed as L_9_ (3^4^) (Table 3) with evaluation scores based on the determination of OA and UA, and the results were then analyzed with variance analysis.

**Table 2:** Factors and levels of the orthogonal array design.

	Factors			
Levels	Amount of honey (A, %)	Temperature (B, °C)	Heating time (C; min)	Moistening time (D; h)
1	50	100	10	2
2	100	120	20	3
3	200	140	30	4

**Table 3:** The L_9_ (3^4^) experiment design of the orthogonal array design.

Factors	Temperature	Heating time	Honey amount	Moistening time	Evaluation score
No. 1	1	1	1	1	93.7517
No. 2	1	2	2	2	94.6764
No. 3	1	3	3	3	84.1200
No. 4	2	1	2	3	84.0749
No. 5	2	2	3	1	82.1996
No. 6	2	3	1	2	97.3241
No. 7	3	1	3	2	82.8741
No. 8	3	2	1	3	100.0000
No. 9	3	3	2	1	90.3395
Mean 1	90.849	86.900	97.025	88.764	
Mean 2	87.866	92.292	89.697	91.625	
Mean 3	91.071	90.595	83.065	89.398	
R	3.205	5.392	13.960	2.861	

### Verification of the method

The method has been verified by randomly testing CPPY and PPPY available in the medicinal market. The test samples were subjected to the methods described above. The data were then analyzed by PCA and OPLS-DA.

## Results

### Determination of OA and UA

The determination of OA and UA in CPPY, HPPY, and PPPY are presented in Table 4. Rankings of the contents in the samples were CPPY ≈ HPPY > PPPY. HPPY and CPPY contained higher OA and UA contents than PPPY, while OA and UA in CPPY were similar to those in HPPY, indicating the chemical stability of OA and UA under heating conditions [27]. Two reasons may explain why HPPY contained more OA and UA than PPPY: 1) PPPY may contain less herb materials than HPPY because half of PPPY is made up of honey, and 2) Maillard reactions may occur during the heating process due to the existence of organic acids and polysaccharides [28]. According to ancient literature, HPPY and PPPY have the same significance in clinical practice but have different pharmacological effects. Interestingly, PPPY is more commonly used than HPPY in clinical practice nowadays. Additionally, HPPY has received less attention than PPPY. Considering the comprehensiveness and complexity of TCM, further investigation is required to determine the pharmacological values of HPPY and PPPY.

**Table 4:** Determination of OA and UA in CPPY, PPPY, and HPPY (N = 3).

	PPPY (%)		HPPY (%)	
Temperature (°C)	OA	UA	OA	UA
80	0.0879	0.4912	0.1313	0.7756
100	0.1138	0.5776	0.1799	0.9190
120	0.1193	0.5711	0.1250	0.7651
140	0.1147	0.5944	1.1892	0.9244
160	0.1102	0.5503	0.1463	0.8081
0 (CPPY)	---	---	0.1723	0.7387

### Tentative peak assignment by UPLC-QTOF/MS

**Figure 1:**
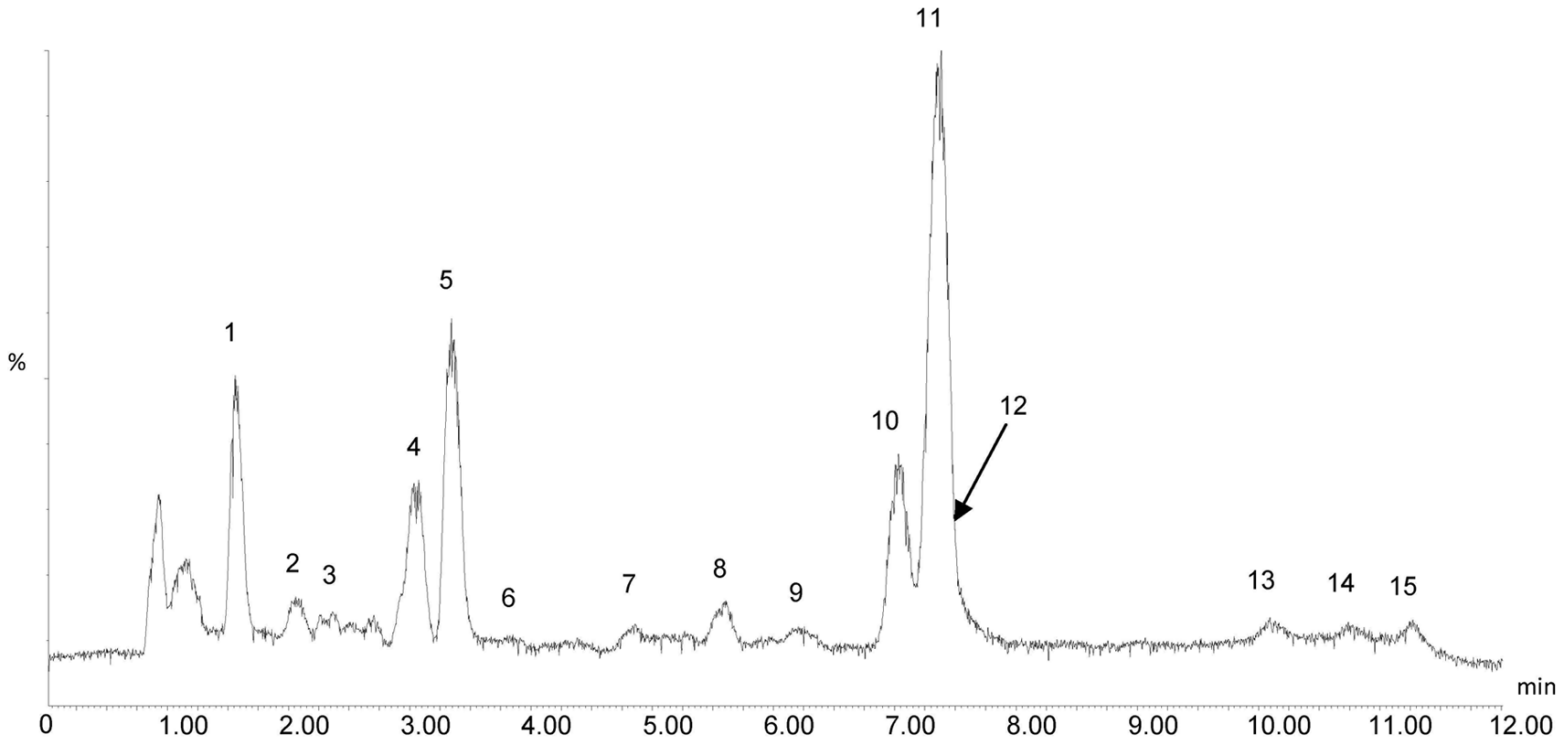
Representative profiling of a PPY sample.

Table 5 lists the tentatively identified compounds in CPPY, HPPY, and PPPY. A total of 15 compounds were identified by UPLC-QTOF/MS based on database interrogation, standard compounds, and references, as shown in Figure 1 [29]–[34]. Peaks 10 and 11 were identified as OA and UA, respectively, based on retention times, MS, and MS/MS fragment ions [28], [33]. Peaks 1, 4, and 5 were identified as euscaphic acid, maslinic acid, and 2α-hydroxyursolic acid, respectively, based on retention times, and MS data for these peaks were consistent with references [29], [34]. Peaks 2, 3, 7, 9, and 12 were identified as 2α,19α-dihydroxyurs-3-oxo-urs-12-en-28-oic acid, 3-O-p-coumaroyltormentic acid, hyptadienic acid, 3β-O-coumaroyl-2α-hydroxy-urs-12-en-28-oic acid, and palmitic acid, respectively, based on molecular mass and MS/MS fragment ions [30], [32], [33]. Linolenic, linoleic, stearic, and isomeric stearic acids were identified according to molecular masses and fragment ions and have been identified in the seeds or fruits of *E. japonica* [31]. All other compounds have been previously reported to be present in the leaves of *E. japonica.*

**Table 5:** Tentatively identified compounds from leaves of *E. japonica.*

				[M–H]^−^ *m/z*				
Peak no.	t_R_ (min)	Assigned identity	Molecular formula	Mean measured mass (Da)	Theoretical exact mass (Da)	ppm	Fragments *m/z*	References
1	1.52	euscaphic acid	C_30_H_48_O_5_	487.3411	487.3423	−2.5	469.3325 [M-H-H_2_O]^−^, 425.3409 [M-H-H_2_O-CO_2_]^−^	[29], [34]
2	1.96	2α, 19α-dihydroxyurs-3-oxo-urs-12-en-28-oic acid	C_30_H_46_O_5_	485.3231	485.3267	−7.4	467.3167 [M-H-H_2_O]^−^, 423.3255 [M-H-H_2_O-CO_2_]^−^	[30]
3	2.31	3-O-p-coumaroyltormentic acid	C_39_H_54_O_7_	633.3813	633.3850	−5.8	487.3415 [M-H-C_9_H_6_O_2_]^−^	[30]
4	2.87	maslinic acid	C_30_H_48_O_4_	471.3431	471.3474	−9.1	427.3688 [M-H-CO_2_]^−^, 409.3485 [M-H-CO_2_-H_2_O]^−^	[29], [34]
5	3.16	2α-hydroxyursolic acid	C_30_H_48_O_4_	471.3431	471.3474	−9.1	427.3583 [M-H-CO_2_]^−^, 409.3485 [M-H-CO_2_-H_2_O]^−^	[29], [34]
6	3.61	linolenic acid	C_18_H_30_O_2_	277.2168	277.2168	0.7	255.23334, 217.0050	[31]
7	5.08	hyptadienic acid	C_31_H_50_O_3_	469.3310	469.3318	−3.6	425.3776 [M-H-CO_2_]^−^	[30]
8	5.20	linoleic acid	C_18_H_32_O_2_	279.2324	279.2324	0.7	255.2333, 217.0050	[31]
9	5.37	3β-O-coumaroyl-2α-hydroxy-urs-12-en-28-oic acid	C_39_H_54_O_6_	617.3852	617.3842	1.6	471.3441 [M-H-C_9_H_6_O_2_]	[32]
10	6.64	oleanolic acid	C_30_H_48_O_3_	455.3505	455.3525	−4.4	411.3620 [M-H-CO_2_]^−^	[29], [34]
11	7.27	ursolic acid	C_30_H_48_O_3_	455.3505	455.3525	−4.4	411.3615 [M-H-CO_2_]^−^	[29], [34]
12	7.45	palmitic acid	C_16_H_32_O_2_	255.2333	255.2324	3.5	217.0050	[33]
13	10.03		C_18_H_36_O_2_	283.2637	283.2637	0	255.2333	[31]
14	10.64		C_18_H_36_O_2_	283.2637	283.2637	0	255.2333	[31]
15	11.19		C_18_H_36_O_2_	283.2637	283.2637	0	255.2333	[31]

### PCA of CPPY, HPPY, and PPPY

PCA uses an N-dimensional vector approach to separate samples on the basis of the cumulative correlation of all metabolite data and then identifies the vector (eigenvector) that yields the greatest separation among samples without requiring prior knowledge of the data sets [35]. Mean-centered and par-scaled (scaled to square root of SD) mathematical methods were performed to pretreat the data sets resulting from the above data. Samples processed using the same conditions were replicated with 3 individuals (N = 3). A total of 1058 variables were used to create the model. The 2-component PCA model cumulatively accounted for 50.51% of variation (PC1, 32.95%; PC2, 17.56%).

**Figure 2:**
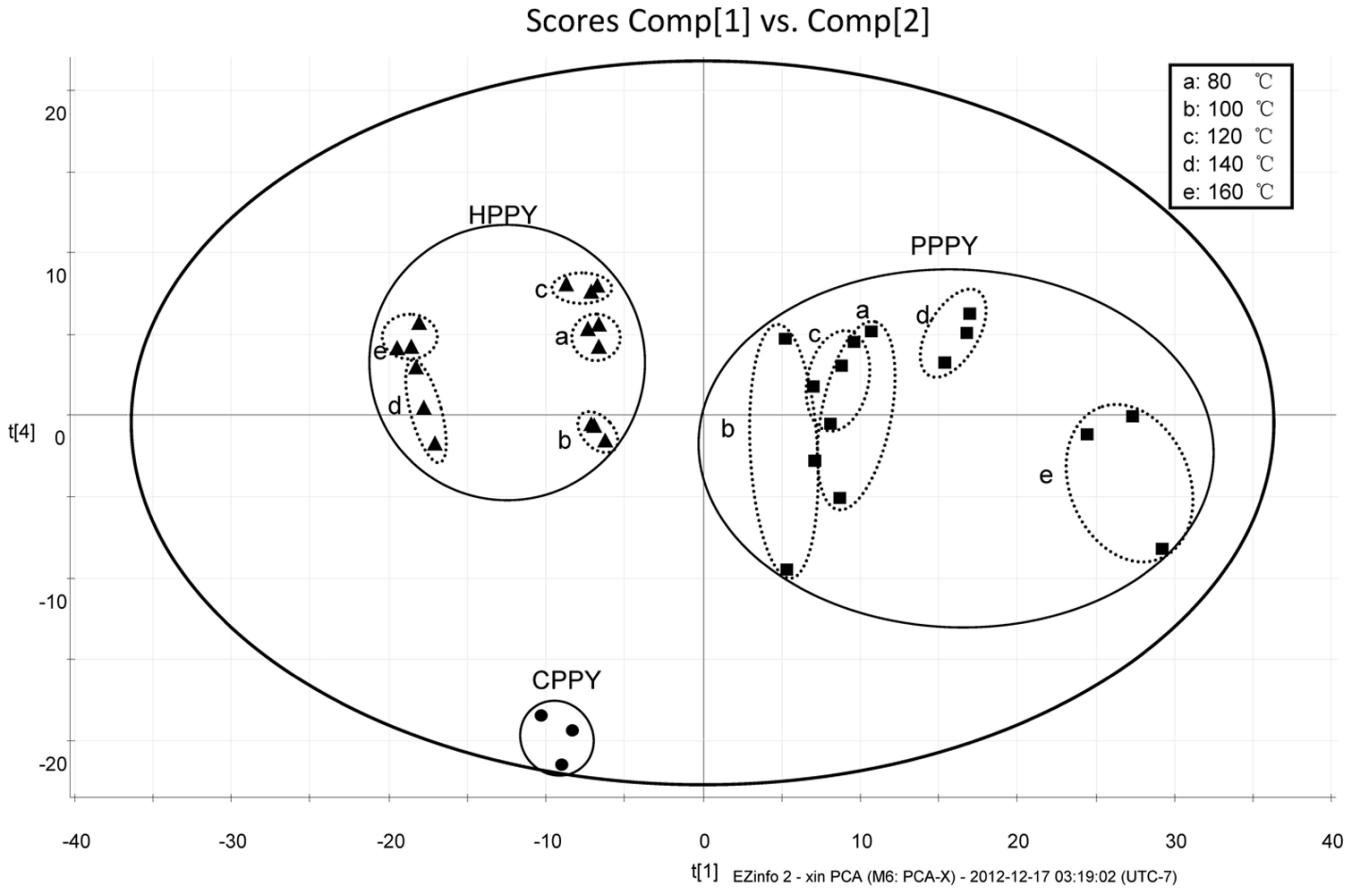
PCA (scores plot) of CPPY, HPPY, and PPPY.

Figure 2 shows that CPPY, HPPY, and PPPY samples were divided into 3 main clusters observed in the PCA scores plot. Such division indicated that use of different processing methods could significantly alter the composition of compounds and that CPPY, HPPY, and PPPY were distinct from each other. This distinct separation could be representative of their multiple pharmacological effects.

The PCA score plot illustrates that samples processed at different temperatures could be clearly discriminated. In HPPY, samples treated at 80, 100, and 120°C differed from those treated at 140 and 160°C. Obviously, the samples changed dramatically after heating at 140°C, and chemical differences between 140 and 160°C treatments were not evident. PPPY samples treated at 80, 100, and 120°C were clustered into 1 group and separated from those samples treated at 140 and 160°C. Unlike HPPY samples, PPPY samples processed at 140 and 160°C were located far from each other. This finding indicated that dramatic chemical changes occurred when the processing temperature was raised to 140°C. In addition, honey treatment may lead to such results. Therefore, this experiment clearly demonstrated the importance of temperature and auxiliary materials, i.e., honey, during processing.

### Orthogonal array design and standardized production of PPPY

The results of the orthogonal array design are shown in Table 2, 3 and 6. Table 3 shows that the optimized production procedure for PPPY was performed in experiment no. 8 (A_3_B_2_C_1_D_3_) depending on the quality scores (based on the determination of OA and UA). Therefore, the optimized production of PPPY was performed with 50% honey at 140°C for 20 min after 4 h of moistening time. Table 6 shows the analysis of variance, demonstrating that the most significantly influential factor was the amount of honey, which had the highest critical *F* value.

**Table 6:** Analysis of variance of the orthogonal array design.

Factors	SS	Degrees of freedom	*F* a
Temperature	19.221	2	1.419
Heating time	45.600	2	3.366
Honey amount	292.594	2	21.598*
Moistening time	13.547	2	1.000
Error	13.55	2	

a The critical *F* value was 21.598 (* *p*<0.05).

The orthogonal array design confirmed the results of the PCA above. The PCA showed that temperatures of 140 and 160°C could significantly alter the process, unlike temperatures of 80, 100, and 120°C. From the visual observation of PPPY, PPPY samples processed at 160°C showed a dark color, indicating an excessive heating process. Considering the results of PCA and the orthogonal array design, we regarded 140°C as the optimal temperature in the processing PPPY. The orthogonal array design experiment demonstrated a certain credibility in the standardization of processed TCMs. Through this experiment, we recommend the A_3_B_2_C_1_D_3_ processing steps for the production of PPPY.

### OPLS-DA and marker identification

To identify markers for the discrimination between crude and processed samples, extended statistical analysis was performed to generate the S-plot of OPLS-DA. In the S-plot, each point represented a t_R_–*m/z* ion pair. The X axis represented the contribution of the ion. The distance of the t_R_–*m/z* ion pair pointed to the origin on the X axis and represented the contribution of this ion to the differences between the 2 groups. The Y axis represented the confidence of the ion. The distance of the t_R_–*m/z* ion pair pointed to the origin on the Y axis and represented the confidence level of this ion. Thus, the t_R_–*m/z* ion pointing to the 2 ends of the “S” represented the characteristic markers with the highest confidence in each group.

**Figure 3:**
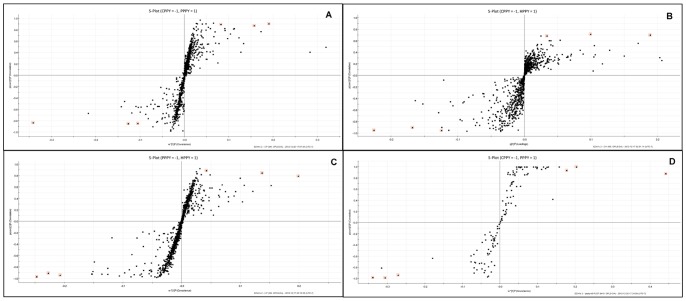
OPLS-DA (S-plot) of PPY samples. (A, CPPY and PPPY from experimental samples; B, CPPY and HPPY from experimental samples; C, HPPY and PPPY from experimental samples; D, CPPY and PPPY from commercial samples).

In Figure 3, pairs of these samples were compared in an S-plot. The circled points were regarded as the highest confidence markers, which could be used as potential points in distinguishing between markers. The results of OPLS-DA showed that UPLC-QTOF/MS could be applied to distinguish between raw and processed PPY by the S-plot (Figure 3). A total of 6, 6, and 6 credible and significant markers are found to be available in distinguishing between CPPY/HPPY, CPPY/PPPY, and HPPY/PPPY samples, respectively (Table 7). Two identities of potential markers b and c (Table 7) were tentatively assigned (Table 5) [29], [30], [34]. The components correlated to these 2 ions were tentatively assigned as 2α-hydroxyursolic acid and 3-O-p-coumaroyltormentic acid. Therefore, significant differences existed between crude and processed PPY according to the S-plot of OPLS-DA, and these credible markers could be considered in distinguishing between and identifying these different samples.

**Table 7:** Marer t_R_–*m/z* ion pairs in the S-plot.

Experimental Loquat leaves						Commercial Loquat leaves	
CPPY/PPPY		CPPY/HPPY		HPPY/PPPY		CPPY/PPPY	
	3.32–471.3449 (**b**)		1.48–293.2056		2.33–633.3749 (**c**)		3.17–471.3453(**b**)
CPPY	0.96–451.0948	CPPY	0.95–341.0990	HPPY	1.57–826.5356	CPPY	1.54–487.3410
	0.93–517.2170		0.93–517.2170		1.56–572.3066		2.87–471.3481
	0.93–503.1484		2.33–633.3749 (**c**)		0.93–503.1484		0.94–341.1066
PPPY	0.95–221.0600 (**a**)	HPPY	2.42–663.3751	PPPY	1.56–667.3883	PPPY	0.94–221.0670 (**a**)
	0.95–539.1216		0.99–331.2377		0.95–221.0600 (**a**)		0.98–191.0561

### Verification test

**Figure 4:**
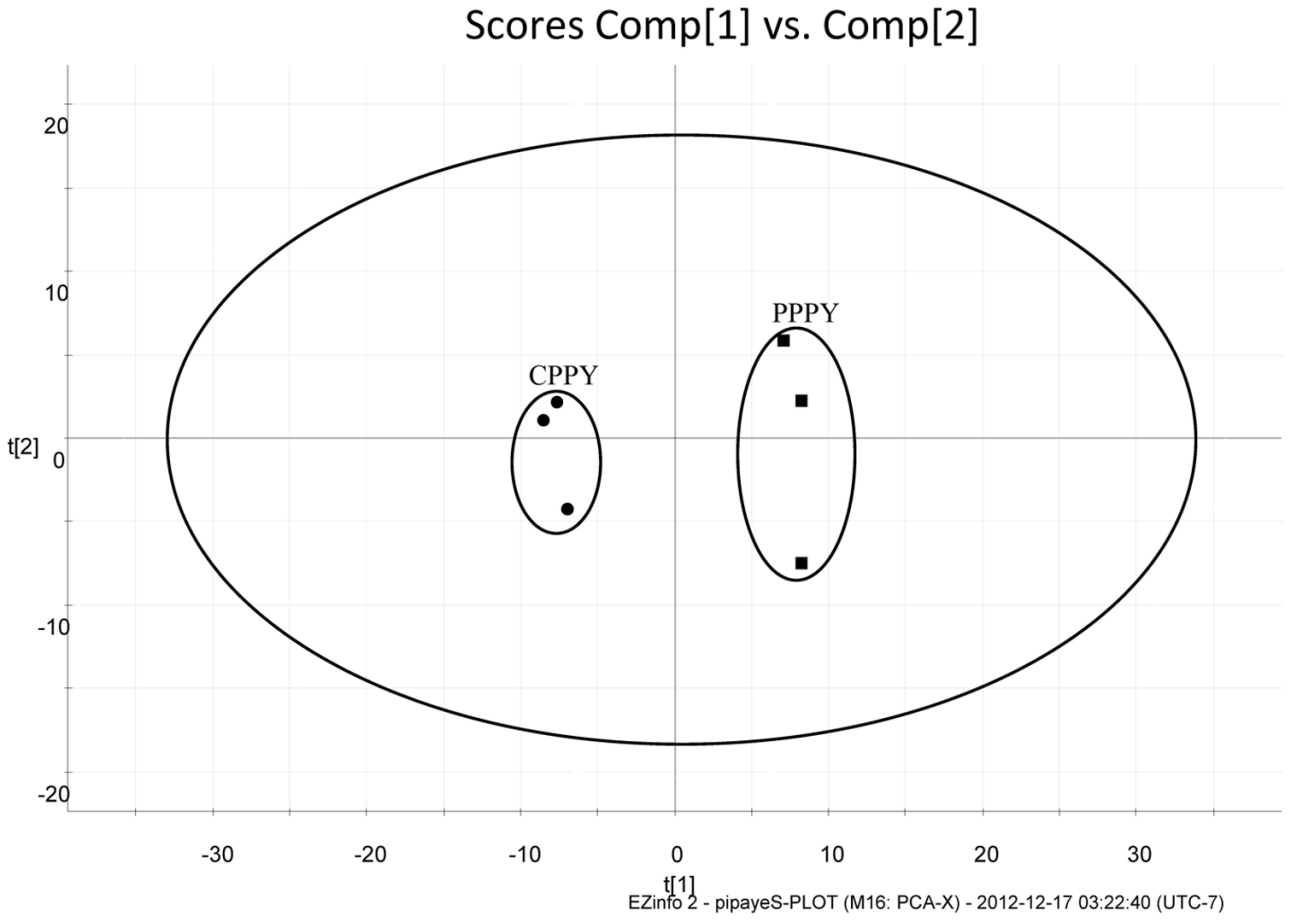
PCA (scores plot) of commercial CPPY and PPPY.

**Figure 5:**
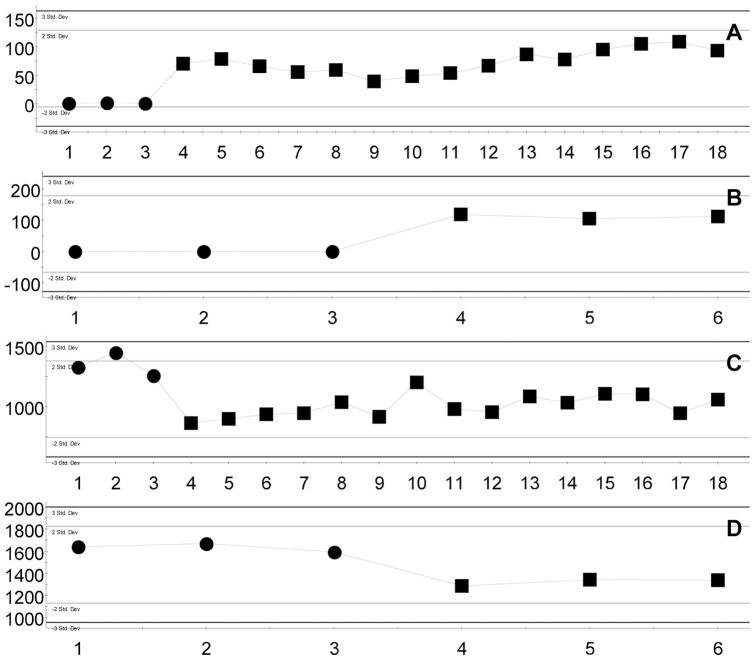
Ion intensities of markers a and b. (•, CPPY; ▪, PPPY. A, marker a in experimental samples; B, marker a in test samples; C, marker b in experimental samples; D, marker b in test samples).

In the verification test, CPPY and PPPY could be separated by the PCA score plot (Figure 4). This demonstrated that UPLC-QTOF-MS could be used as the method for identification between commercial CPPY and PPPY. Additionally, OPLS-DA was performed to generate an S-plot (Figure 3D). Two marker ions, marker a (0.95_221.0600/0.94_221.0670) and marker b (3.32_471.3449/3.17_471.3453), were successfully verified. Marker a could be detected in experimental and test PPPY samples, but could not be detected in CPPY (Figure 5A, 5B). The ion intensity of marker b in CPPY was higher than that in PPPY in experimental samples, and this was verified in test samples (Figure 5C, 5D). These results verified that markers a and b could be used as indicators in distinguishing between CPPY and PPPY.

## Conclusion

This investigation explored the processing technology of Loquat leaves by UPLC–QTOF/MS and chemometrics. PCA successfully illustrated the differences in samples processed using different processing methods. We were able to distinguish between CPPY, HPPY, and PPPY, and the differences between samples processed at different temperatures were also presented, which indicated the dramatic differences caused by processing methods. OPLS-DA identified 2 unique marker ions that could discriminate between CPPY and PPPY, for the first time. This finding was verified by experiments using test samples. The optimized processing condition used 50% honey at 140°C for 20 min after 4 h of moistening time, in an orthogonal array design. This investigation provides insights into the development of processing technology in TCM.
